# Prehospital Flumazenil: Five-Year Review of Use, Indications, Outcomes, and Adverse Events in Suspected Benzodiazepine Overdose

**DOI:** 10.5811/westjem.62063

**Published:** 2026-07-31

**Authors:** Cameron G. Hanson, Bryan P. Beaver, Eric Frantz, Thomas Dunn, Stephen Thornton, Isabel Cava, Ryan C. Jacobsen

**Affiliations:** * The University of Kansas Medical Center, Department of Emergency Medicine, Kansas City, Kansas; † Kansas City University, College of Osteopathic Medicine, Kansas City, Missouri

## Abstract

**Introduction:**

Historically, flumazenil was employed as both a diagnostic aid in unresponsive patients and as an antidote for suspected benzodiazepine overdose in both emergency departments and out-of-hospital settings. However, its prehospital application has been historically constrained by long-standing concerns regarding adverse events, leading many emergency medical service (EMS) systems to restrict or avoid its use. The use of flumazenil in the prehospital setting remains underreported in the literature, despite its inclusion in the treatment protocols of various EMS agencies. We conducted a descriptive analysis of the use of flumazenil by EMS in the out-of-hospital setting across the state of Kansas over a five-year period. We characterize the frequency, indications, patient outcomes, and associated adverse events of flumazenil administration, thereby contributing to a better understanding of its role, safety, and clinical relevance in prehospital care.

**Methods:**

We performed a retrospective observational cohort design to evaluate the use of flumazenil by EMS clinicians in the state of Kansas from January 2015–August 2020. Patient care reports documenting prehospital administration of flumazenil were obtained from the Kansas Board of EMS. Data extracted included demographics, indications, initial and subsequent Glasgow Coma Scale (GCS) scores when available, clinical response, and the occurrence of adverse events. Our primary outcome measure was GCS improvement following administration, with secondary outcome measures including patterns and outcomes with flumazenil use in the prehospital setting, clinical response, and occurrence of adverse side effects.

**Results:**

A total of 80 cases involving EMS administration of flumazenil were identified with 69 cases meeting inclusion criteria. No seizures or adverse events were associated with flumazenil use (0/69; 95% CI, 0–4.3%), even in administrations with possible contraindications. Of the 69 cases analyzed, 24 cases documented suspected benzodiazepine overdose, all of which documented neurologic improvement (24/24; 95% CI, 87.5–100%), with 17 receiving flumazenil alone and 7 with naloxone coadministration.

**Conclusion:**

In this small sample, no adverse events were noted with flumazenil administration in the prehospital setting. Flumazenil administration was commonly associated with documented improvement in GCS in suspected benzodiazepine overdose. These findings suggest that exclusion in EMS protocols based solely on concern for adverse events in the prehospital setting can be reconsidered. Further research is needed to better define its safety, efficacy, and clinical utility in this setting.

## INTRODUCTION

Flumazenil is an imidazobenzodiazepine and a selective GABA_A_ receptor antagonist that acts through competitive inhibition at the benzodiazepine binding site. It is approved by the U.S. Food and Drug Administration (FDA) for the reversal of benzodiazepine-induced sedation during procedural sedation and general anesthesia in both adult and pediatric patients. It is also indicated for the treatment of benzodiazepine overdose in adults. Flumazenil can be administered through multiple routes, including intravenous, intramuscular, and intranasal delivery. It provides rapid and consistent reversal of the pharmacologic effects of benzodiazepines.^1^ In addition to reversing the effects of benzodiazepines, flumazenil can also antagonize the sedative effects of certain nonbenzodiazepine agents that act on the same benzodiazepine binding site of the GABA_A_ receptor. This includes drugs such as zolpidem, a nonbenzodiazepine hypnotic commonly used for the treatment of insomnia, which shares similar receptor-binding characteristics despite structural dissimilarity.^2^ Due to its rapid onset and relatively short duration of action, flumazenil was initially regarded as an ideal antidote for benzodiazepine overdose. It was considered safe, lacking intrinsic agonist activity, and was recommended both for reversing benzodiazepine-induced coma and as a diagnostic agent to identify benzodiazepine involvement in comatose patients, both in emergency departments (ED) and out-of-hospital settings.^3^,^4^

However, concerns soon emerged in the medical literature regarding the potential for flumazenil to precipitate acute benzodiazepine withdrawal and provoke seizures. The actual incidence and clinical significance of these adverse effects remain a topic of debate. Notably, flumazenil has also been investigated as a therapeutic option for managing benzodiazepine withdrawal; most seizure cases associated with its use have been reported in the context of polydrug ingestions rather than isolated benzodiazepine exposure.^5^–^7^ Widespread concerns about the risk of precipitating seizures and benzodiazepine withdrawal led to numerous warnings and clinical cautions against the use of flumazenil, particularly in patients with a history of seizure disorders, chronic benzodiazepine use, or suspected polydrug ingestion.^8^,^9^

Despite the shift toward more conservative use, flumazenil continues to be administered in hospital settings, EDs, and emergency medical service (EMS) systems, with reports supporting both its effectiveness and safety.^10^–^12^ However, its role in the prehospital environment remains underexplored, with very few studies published specifically addressing flumazenil use in out-of-hospital care.^13^ This question has become more pertinent due to increasing abuse of benzodiazepines.^14^ In this descriptive study we aimed to characterize the use of flumazenil by state-wide EMS clinicians. The primary outcomes were adverse reactions after administration and clinical effect when given for suspected benzodiazepine overdoses.

**Population Health Research Capsule**

What do we already know about this issue?

*Flumazenil reverses benzodiazepines but is rarely used prehospital due to seizure risk concerns, with limited emergency medical services (EMS) data on safety and outcomes*.

What was the research question?

*What are flumazenil use patterns by EMS, Glasgow Coma Scale (GCS) outcomes, and adverse event rates in prehospital care?*

What was the major finding of the study?

*There were 0/69 adverse events (95% CI, 0–4.3%); GCS improved in 24/24 suspected benzodiazepine overdoses (95% CI, 87.5–100%)*.

How does this improve population health?

*These findings support safe EMS flumazenil use, enabling faster reversal of benzodiazepine overdose, improved consciousness, and potentially reduced morbidity*.

## METHODS

### Design

This retrospective observational cohort study evaluated flumazenil administration by EMS clinicians across the state of Kansas from January 1, 2015–August 31, 2020.The study objective, population, and inclusion and exclusion criteria were defined a priori. We obtained data from the Kansas EMS Information System (KEMSIS), a statewide National EMS Information System version 3–compliant repository managed by the Kansas Board of EMS. This database captures EMS response data statewide from 2015 onward and, as of October 1, 2019, included approximately 98% of total EMS call volume in Kansas. Deidentified, redacted EMS patient care reports were provided by the Kansas Board of EMS upon request.

This study was designed and reported in accordance with methodological recommendations for emergency medicine chart review studies as described by Worster and Bledsoe. Data elements and outcomes were specified a priori. All data were abstracted from standardized EMS patient care reports using predefined variables, and the study cohort was constructed with transparent accounting of exclusions. We excluded cases for documentation errors or if flumazenil administration occurred outside the prehospital setting.

Extracted variables included patient demographics, documented indication for flumazenil use, pre- and postadministration Glasgow Coma Scale (GCS) scores, narrative clinical response (eg, improvement in GCS, seizure-like activity), vital signs, dose and route of administration, relevant medical history, and reported adverse events. Contraindications to flumazenil use were defined as the presence of focal neurologic deficits, seizure-like activity, or suspected ingestion of nonbenzodiazepine substances, including tricyclic antidepressants, other seizurogenic medications, or polysubstance exposures.

We defined adverse events as development of any new symptom following administration or worsening of any preexisting symptoms. Improvement in GCS was defined as an increase of GCS by at least one point based on explicit documentation or through narrative interpretation. Proportions are presented with 95% confidence intervals calculated using the exact Clopper–Pearson method for binomial data, given the small sample sizes and presence of proportions near 0% and 100%.

### Subjects Selection Criteria

#### Inclusion Criteria

We included all EMS patient care reports from participating agencies documenting the administration of flumazenil by EMS clinicians.

#### Exclusion Criteria

We excluded cases in which flumazenil was administered outside the out-of-hospital environment, including those occurring within hospital facilities or during interfacility transfers not classified as prehospital care (ie, given by an EMS clinician while physically located at the sending or receiving facility, or given by a non-EMS clinician) were excluded. Additional exclusion criteria included age < 18, cases with missing data (ie, no initial GCS, missing indication, etc).

#### Sample Size

A total of 80 EMS patient care reports documented flumazenil administrations. Of these, 11 cases were excluded based on the exclusion criteria above including the following: nine interfacility transfers where flumazenil was administered at the sending facility prior to EMS involvement; one case of administration by an ambulatory surgery clinic before EMS arrival; and one case excluded due to a documentation error where fentanyl administration was incorrectly recorded as flumazenil. The final study sample included 69 patients.

## RESULTS

After applying exclusion criteria, 69 cases involving the administration of flumazenil by EMS personnel in the out-of-hospital setting were identified from the available patient care reports. These cases included interfacility transfers classified as prehospital care, suspected or possible nonbenzodiazepine overdose (ie, suspected opioid overdose, or type of overdose not specified), suspected benzodiazepine overdose (as specified in the patient care report), altered mental status of unknown etiology, and cardiac arrest (Table).

Of the 69 total prehospital flumazenil administrations analyzed, no patients who received flumazenil developed any new or worsening symptoms (0/69; 95% CI, 0–4.3%). Crucially, this also includes several administrations in cases with possible contraindications including one patient with focal neurologic deficits; two with seizure-like activity; two with tricyclic antidepressant coingestion, three with known anticholinergic overdose, and 14 other polydrug ingestions.

Of the 24 cases that documented suspected benzodiazepine overdose, all patients demonstrated clinical improvement following flumazenil administration (24/24; 95% CI, 87.5–100%). Improvement was observed in all patients receiving flumazenil alone (17/17; 95% CI, 82.4–100%) and in those receiving flumazenil with naloxone (7/7; 95% CI, 57.1–100%).

Of the seven cases in which GCS improvement was reported after coadministration of flumazenil and naloxone, two of these documented specifically no response to naloxone but improvement following flumazenil (2/7; 95% CI, 3.7–71.0%). Among the 17 cases attributed to flumazenil alone, 13 involved patients with an initial GCS < 8 who experienced documented improvement postadministration by at least one point. The remaining four cases had documented GCS scores > 8 prior to flumazenil administration and saw improvement by at least one point following administration (Figure).

These findings suggest that when EMS clinicians suspected benzodiazepine overdose in the prehospital setting, flumazenil administration were more frequently associated with clinical improvement. Among the remaining EMS-administered flumazenil cases (those documenting suspected nonbenzodiazepine overdose, altered mental status of unknown etiology, cardiac arrest, and interfacility transfers classified as prehospital care), several yielded notable outcomes. Two patients with “altered mental status of unknown origin” experienced improved GCS post-administration (2/16; 95% CI, 1.6–38.3%). One administration was given for cardiac arrest, resulting in the return of spontaneous circulation postadministration (1/10; 95% CI, 0.3–44.5%). In three cases with contraindications, including two cases with seizure-like activity (documented indication of “altered mental status of unknown etiology”), and one with confirmed amitriptyline ingestion, flumazenil administration was associated with improvement in GCS.

## DISCUSSION

Overall, there is a significant lack of data regarding the use of flumazenil in the prehospital setting. To our knowledge, this is the first study of its kind to examine adverse effects of prehospital flumazenil use. In a 2025 systematic review on adverse events of both intramuscular and intravenous flumazenil, the study authors noted that due to the lack of quality evidence, especially in the prehospital setting, a conclusion on flumazenil’s overall safety is difficult to make.^15^ However, in their review, which included one clinical study examining intramuscular flumazenil, and two systematic reviews examining parenteral flumazenil, along with multiple animal studies, overall rates of adverse events were low. Their systematic review also reported that seizures occurred in < 2% of patients receiving flumazenil, with no patients developing refractory status epilepticus, and no reported deaths.

Additionally, prior studies have suggested that the risk of seizures may be dose-dependent, with a higher likelihood observed at doses > 1 mg.^15^–^17^ Among the 69 cases analyzed in our study, the average flumazenil dose was 0.8 mg, with a median of 0.4 mg, a mode of 0.2 mg, and a maximum dose of 7.5 mg. The most frequent pattern of dosing was observed to be an initial dose of 0.2, most commonly given twice. The most common route of administration was intravenous (51/69; 95% CI, 63.5–84.3%), with intramuscular the next most frequently seen (18/69; 95% CI, 15.7–36.4%). No cases documented intranasal administration. As no adverse events were recorded, we were unable to observe any association with adverse events and dosing and/or route of administration.

Rates of opioid overdoses appear to be down trending in the United States; however, rates of benzodiazepine use have been increasing, especially since the COVID-19 pandemic.^18^–^19^ As with all respiratory depressants, a concern for benzodiazepine overdose is airway management. In theory, flumazenil can help reduce the need for advanced airway management by improving a patient’s mental status. This benefit might have been seen in our study, as 15 patients with suspected benzodiazepine overdose with initial GCS <. 8 saw an improvement in GCS that otherwise may have required advanced airway management. This concept may be more relevant in rural settings where patients with advanced airways may require prolonged transport times or result in automatic transport to higher level of care centers.

When EMS clinicians documented the indication for flumazenil as “suspected benzodiazepine overdose,” a high proportion of patients demonstrated clinical improvement, with 19 of 24 cases (79%; 95% CI, 58%–93%) showing an increase in GCS attributed to flumazenil administration. This finding may highlight the potential value of accurate prehospital recognition of benzodiazepine toxicity when flumazenil is used within EMS protocols, and it underscores the importance of targeted clinician education to support appropriate patient selection.

However, flumazenil is available in only a minority of EMS systems in the United States and is rarely administered in the prehospital setting. This may be because flumazenil has a typical shelf life of approximately 18–36 months (most commonly ~24 months) when stored at controlled room temperature. Also, the average acquisition cost is approximately $10–$25 per 1 mg vial in U.S. hospital purchasing systems. Given this combination of limited shelf life and the expense of maintaining a medication with infrequent prehospital use, many EMS agencies elect not to include flumazenil in their standard formulary. These considerations are reasonable given the infrequent use of flumazenil in the prehospital environment, along with its limited shelf life and the associated costs of maintaining inventory. However, the findings from the present study suggest that exclusion of flumazenil from EMS formularies primarily due to concerns regarding adverse reactions may warrant reevaluation. In our cohort, clinical improvement was commonly observed when flumazenil was administered in cases of suspected isolated benzodiazepine toxicity, supporting the potential utility of more selective, protocol-guided use in appropriately identified patients.

## LIMITATIONS

Our study has several limitations. Firstly, the retrospective design is inherently limited as it restricts the ability to establish control for confounding variables. Additionally, the reliance on EMS documentation may have inaccuracies or missing data. As most EMS documentation is not done in real time, it is difficult to know whether benzodiazepine overdose was truly suspected prior to administration, or whether it was identified in retrospect. Similarly, nearly all patient reports did not include time to effect after flumazenil administration, length of transport time, or whether any adverse reactions were experienced after the patient was handed off from EMS care. Our sample size is also relatively small, which limits statistical power and subgroup analysis. In the case of coadministration of naloxone, only two cases documented a flumazenil-specific effect on GCS. Our study also was limited with respect to obtaining more current data. Requests to KEMSIS for updated data could not be completed by publication. Neither could we obtain information on flumazenil protocols for specific agencies.

Future studies would ideally look at matched cohorts comparing outcomes in patients with suspected benzodiazepine overdose who did and did not receive flumazenil, including the need for airway intervention, seizure occurrence, and GCS change.

## CONCLUSION

Emergency medical service protocols are often necessarily adapted to local patient populations, operational resources, and system-level training requirements. From a formulary perspective, flumazenil presents practical challenges, including a moderately finite shelf life and a relatively high per-vial acquisition cost within U.S. hospital purchasing systems. In combination with its infrequent deployment in the prehospital setting, these factors provide a rational basis for many EMS systems to exclude it from routine stocking.

Despite these considerations, the present analysis raises questions about whether noninclusion based largely on anticipated low utilization may be overly conservative in select systems. More importantly, this study suggests that exclusion of flumazenil from EMS formularies primarily due to concerns regarding adverse reactions may warrant reevaluation. These findings contribute to a limited but growing body of literature describing outcomes associated with EMS use in carefully selected clinical scenarios.

Taken together, these results may support reconsideration of flumazenil as a selectively available option within EMS protocols, particularly in systems with robust clinical governance and well-defined indications for use.

## Figures and Tables

**Figure:**
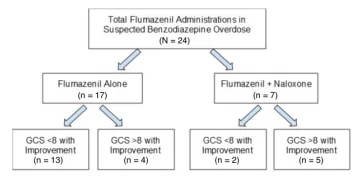
Changes in Glasgow Coma Scale with flumazenil administration in suspected benzodiazepine overdose in a study evaluating the use of flumazenil by emergency medical services clinicians. *GCS*, Glasgow Coma Scale.

**Table:** Characteristics of emergency medical services flumazenil administrations (n = 69) in a study evaluating the use of flumazenil by emergency medical services clinicians.

Category	n	%
Interfacility transfers classified as prehospital care	5	7%
Suspected/possible non-benzodiazepine overdoses	14	20%
Suspected benzodiazepine overdoses (documented)	24	35%
Altered mental status of unknown etiology	16	23%
Cardiac arrest	10	14%
Return of spontaneous circulation achieved	1	0.01%
